# Pathways linking relative deprivation to blood pressure control: the mediating role of depression and medication adherence among Chinese middle-aged and older hypertensive patients

**DOI:** 10.1186/s12877-023-03769-6

**Published:** 2023-01-31

**Authors:** Wenzhe Qin, Lingzhong Xu

**Affiliations:** 1 Center for Health Management and Policy Research, School of Public Health, Cheeloo College of Medicine, Shandong University, Jinan, 250012 China; 2 National Health Commission (NHC) Key Lab of Health Economics and Policy Research, Shandong University), Jinan, 250012 China

**Keywords:** Relative deprivation, Blood pressure control, Depressive symptoms, Medication adherence, Mediating effect

## Abstract

**Background:**

Studies have demonstrated that individuals of low socioeconomic status have higher blood pressure. Yet, whether socioeconomic inequality would influence blood pressure control and the underlying mechanisms associated with socioeconomic inequality in blood pressure control are unknown. Central to socioeconomic inequality is relative deprivation. We aim to examine the association between relative deprivation and blood pressure control and to investigate the pathways of the association among middle-aged and older adults with hypertension.

**Methods:**

Data were collected from the 2020 Household Health Interview Survey in Taian City, Shandong province. This study included 2382 eligible respondents aged 45 years and older with a diagnosis of hypertension. Our primary outcome was dichotomous blood pressure control. Relative deprivation was calculated with the Deaton Index. Depressive symptoms and medication adherence were considered as mediators. Multivariable binary logistic regression models were used to estimate the effect of relative deprivation on blood pressure control. The “KHB-method” was used to perform mediation analysis.

**Results:**

Among 2382 middle-aged and older adults with hypertension, the mean age was 64.9 years (SD 9.1), with 61.3% females. The overall proportion of participants with uncontrolled blood pressure was 65.1%. Increased relative deprivation was likely to have higher odds of uncontrolled blood pressure (OR: 2.35, 95%CI: 1.78–7.14). Furthermore, depressive symptoms and medication adherence partially mediated the overall association between relative deprivation and blood pressure control, with depressive symptoms and medication adherence explaining 5.91% and 37.76%, respectively, of the total effect of relative deprivation on blood pressure control.

**Conclusions:**

Individual relative deprivation could threaten blood pressure control among middle-aged and older hypertension patients through the mechanisms of depression and medication adherence. Hence, improving blood pressure control may require more than just health management and education but fundamental reform of the income distribution and social security system to narrow the income gap, reducing relative economic deprivation. Additionally, interventions tailoring psychological services and medication adherence could be designed to reduce the harmful effect of relative deprivation on blood pressure control among disadvantaged individuals.

## Background

Hypertension is the most critical risk factor for cardiovascular disease and premature death worldwide [1, 2]. The prevalence and enormous public health burden of hypertension are rising globally, especially in low and middle-income countries [3]. The China Nationwide Hypertension Survey (2012–2015) indicated that 23.2% of Chinese people ≥ 18 years of age (≈244.5 million individuals) had hypertension [4]. Blood pressure control is an essential strategy to reduce hypertension and related cardiovascular disease, prolong life expectancy and enhance patients' quality of life [5]. Uncontrolled hypertension has been estimated to cause 750,000 deaths from cardiovascular disease annually [6]. Blood pressure control is a national public health priority in China, and the government has provided widespread effective therapies and management strategies [7]. However, a persistent gap remains between stated public health targets and achieved blood pressure control rates. Hypertension is estimated to affect about 45% of Chinese people aged ≥ 35 years in China, of whom only 7.2% achieved blood pressure control of < 140 mm Hg systolic and < 90 mm Hg diastolic [8].

Several factors contribute to blood pressure control, and socioeconomic status is the repeatedly mentioned one. Prior observational studies have shown that hypertension is more common and poorly controlled among lower versus higher socioeconomic status groups, with the lack of access to appropriate treatment [9, 10]. However, one study exploring the associations of household income with blood pressure control within a randomized, double-blinded clinical trial found that participants in the lowest-income areas still had poorer blood pressure control and worse outcomes [11]. This study indicated that when the lack of access to appropriate hypertension therapies is no longer a barrier, the relationships between low socioeconomic status and poor blood pressure control persist. Such disparities have motivated researchers to shift their focus away from objective living conditions, including the absolute level of income, and toward study that considers income inequality as a potential risk factor for poor blood pressure control [10].

Within the field of population health, relative deprivation resonated strongly among researchers investigating the impact of income disparity on health, and has been proposed as one mechanism explaining the observed associations between income inequality and adverse health outcomes [12]. Relative deprivation is defined as one's income or socioeconomic status relative to other members in their reference group [13]. Central to this concept is social comparisons triggered by unequal distribution of resources, which can affect individuals' health-related behaviors and outcomes, such as cardiovascular diseases, suicide risk, smoking, and self-rated health [14–17]. The AGES cohort study indicated that relative deprivation increases health risk of cardiovascular disease [18]. Another study in Australia and New Zealand found that people who were relatively more deprived in socioeconomic terms experience higher prevalence of stroke, hypertension and diabetes [19]. However, studies determining the direct and indirect effect of relative deprivation on blood pressure control are scant.

According to the relative deprivation theory, there may be several potential explanations for the association between relative deprivation and health: material pathway, psychosocial pathway, and behavioral pathway [20]. In the material pathway, health inequalities result from worse material/structural living conditions or financial issues, which are often found among socioeconomically deprived people [21]. Moreover, relative deprivation restricts one's access to healthcare resources and services necessary to maintain health [22]. In the psychosocial pathway, increased inequality causes shame, dissatisfaction, and stress among people who are relatively deprived, resulting in adverse health consequences [23]. Our previous study found that depressive symptoms mediated the association between relative deprivation and quality of life [24]. Also, depressive symptoms were proved to increase the risk of hypertension incidence [25]. The presence of depressive symptoms was associated with poor blood pressure control—70% of those with depressive symptoms had uncontrolled hypertension compared with 21% of those without depressive symptoms [26]. The health-related behavioral pathway also explains the relationship between relative deprivation and different health outcomes. Previous studies have demonstrated that relative deprivation contributes to disparities in health behaviors, such as smoking, alcohol consumption, and physical exercise, particularly adherence to treatment regimens [16, 27]. Studies showed that economic deprivation was significantly associated with primary non-adherence to medication and healthy eating dietary goals [28, 29]. As one of the health behaviors, medication adherence plays a specific and critical role in blood pressure control. Higher rates of non-adherence to hypertension treatment were strongly associated with uncontrolled blood pressure [30]. At the same time, limited access to healthcare services and psychological stress from relative deprivation combined result in adverse hypertension outcomes via health behaviors [31]. As the psychosocial and behavioral pathways could represent distinct policies and interventions to increase blood pressure control rates among hypertension patients, it is necessary to examine the existence and significance of the two pathways by conducting evidence-based studies. Our study mainly considered the psychosocial and behavioral pathways in analysis, represented by depression and medication adherence.

China has experienced rapidly increased income inequality since promulgating the economic reform and open-door policy in the late 1970s. China was found to have the joint highest inequality in Asia, and the Gini coefficient for per capita household income was 0.46 in 2015 [32, 33]. After completing the arduous task of eliminating extreme poverty, addressing relative income inequality has become a more pressing issue in China. Income inequality reflects historical and contemporary political factors and powerfully shapes the living conditions of individuals and communities [34]. Several studies suggested that people living in contexts with high levels of inequality over time are more likely to experience worse health outcomes [35, 36]. Understanding the association between inequality and health outcomes is crucial for developing policies to promote public health.

Accordingly, this present study sought to (1) examine the association between relative deprivation and blood pressure control in middle-aged and older adults with hypertension; (2) investigate the mediating roles of depressive symptoms and medication adherence in the association between relative deprivation and blood pressure control. We hypothesized that a higher level of relative deprivation was likely to associate with higher odds of uncontrolled blood pressure; emotional discomfort and unhealthy behavior caused by relative deprivation manifest through depressive symptoms and poor medication adherence, leading to a detrimental impact on blood pressure control.

## Methods

### Study design and data collection

The 2020 Household Health Interview Survey is a cross-sectional, community-based, and observational investigation designed to explore the health status and health service demand and utilization of Chinese adults. This survey was conducted in Taian City, Shandong Province, China. Details on the survey design can be found in our previous study [24]. Samples were selected by a three-stage, stratified sampling design, covering 160 village-level units, and 8654 participants were interviewed. Data were collected during an in-home interview using a paper questionnaire. The questionnaire offers a wide range of information, such as individual demographics, household characteristics, socioeconomic status, and physical and mental health.

In the present study, our analyses were restricted to 2382 eligible respondents aged 45 years and older with hypertension diagnosed by doctors (International Classification of Diseases, 10th Revision, Clinical Modification [ICD-10-CM] code I10) and who have been advised to take antihypertensive drugs. Each participant provided informed consent. The study protocol was approved by the Ethical Committee of the Centre for Health Management and Policy Research, Shandong University (approval number: LL20191220).

## Measurements

### Blood presssure

Blood pressure levels were measured in the participants' homes by primary care providers trained in the study procedures using automated sphygmomanometers (OMRON 7 series; OMRON Healthcare, Inc). The blood pressure before taking medicine is close to the actual value. Therefore, the blood pressure levels were measured in the participants' homes at 7–8 a.m., and try to ensure that the patient's blood pressure was measured before taking the antihypertensive drugs. Readings were obtained after 5 min of seated rest. Three blood pressure measurements were obtained at 30-s intervals [37]. The mean of all available measurements was used to define the systolic and diastolic blood pressure levels.

The study subjects were categorized into two groups according to blood pressure levels: controlled and uncontrolled blood pressure. The latter was defined as systolic blood pressure ≥ 130 mm Hg or diastolic blood pressure ≥ 80 mm Hg for patients with diabetes mellitus, coronary heart disease, or renal disease, and systolic blood pressure ≥ 140 mm Hg or diastolic blood pressure ≥ 90 mm Hg for all others [38, 39].

### Relative deprivation

From the concept of relative deprivation in this study, we shall focus on relative income deprivation. Income should be considered as an indicator of a person’s ability to consume commodities; each unit of income reflects a different bundle of commodities that he can consume [40]. Many studies have demonstrated that relative income deprivation may lead to socioeconomic health disparities [41, 42].

This study uses Deaton formulation to measure objective relative deprivation [13]. Deaton formulation is the variation of the Yitzhaki index, which suggests scaling the Yitzhaki index to the mean income of the total sample in the reference group [40]. In brief, the Deaton formulation captures the proportion of total reference group income obtained by higher-income individuals instead of the sum of their absolute incomes. Deaton formulation is more sensitive to income distribution. It can capture upward and downward comparisons to the referenced individuals and make the index dimensionless. The Deaton formulation is as follows:

$${\mathrm{Deaton}}_{i}=\frac{1}{\mu N}\sum_{j}\left({y}_{j}-{y}_{i}\right){I}_{ij}, {I}_{ij}=\left\{\begin{array}{c}1,if{y}_{j}>{y}_{i} \\ 0,if{y}_{j}\le {y}_{i}\end{array}\right.$$

where *N* is the reference group size of individual *i*. $\mu$ is the mean income in the reference group. ${y}_{i}$ is the individual *i*’s income, and ${y}_{j}$ are all others with higher incomes than individual *i* within that individual’s reference group. Deaton values range from 0 to 1, with a higher score representing a higher relative deprivation.

The income used to calculated Deaton value was the individual’s average annual income in the past year. However, in China’s current cultural background, especially in rural areas and some self-employed workers, it is hard to quantify how much each family member contributes to family finance. Therefore, data on gross annual household income (farming/fishing/livestock income, earnings from employment, retirement wages, investment income, transfer payment of all household members, benefits, and other types of subsidies income) from all household members were collected in our survey. To determine individual income, equivalized income was calculated for each participant by dividing their gross annual household income by the square root of their household size [43].

Also very important is the choice of the reference group when defining relative deprivation. During the investigation, we did not inquire about the participant’s reference group (i.e., whom each individual compared to in terms of income or wealth). Previous studies suggested that income-based social comparisons between individuals were based on similar education level, same age group, and same location [44]. The present study defines reference group as combinations of gender + age group (45–59, 60–74, 75 and above) + educational level (less than primary school, junior high school, senior high school and above) + location (rural, urban). The total number of reference groups under this categorization is 36. People of the same gender, age group, educational level and location becomes a reference group. For example, it was assumed that a 65 years old woman living in rural with primary school education would compare her income to other women with the same attributes instead of drawing comparisons to a 50 years old man living in urban with high school education.

### Depressive symptoms

Depressive symptoms during the past two weeks were assessed using the Patient Health Questionnaire-9 (PHQ-9) [45]. The PHQ-9 is an instrument designed for primary care, either to make a probable diagnosis of major depressive disorder or to continuously measure depressive symptoms. The questionnaire consists of 9 items, and each item has a four-point rating scale (Not at all = 0, Several days = 1, More than half the days = 2, Nearly every day = 3). The total score ranges from 0 to 27, with higher overall scores indicating more severe depressive symptoms. The Cronbach’s alpha coefficient is 0.823 for the PHQ-9 in this study.

Because the optimal cut-off score for diagnosing major depressive disorder with the PHQ-9 remains unclear [46], we use the PHQ-9 as a continuous score to reflect the number of depressive symptoms an individual experienced in the past two weeks prior to the survey. This linear specification avoids a binary categorization and allows subthreshold depression to be evaluated. Subthreshold depression is characterized as clinically relevant depressive symptoms, without meeting the criteria for a full-blown major depressive disorder, with a considerable impact on the quality of life and a large-scale economic burden [47]. Referring to previous literature [48–50], which used PHQ-9 to assess depressive symptoms, we conducted the analyses using the PHQ-9 score as a continuous scale and used mean(Standard deviation) to conduct descriptive statistics.

### Medication adherence

Medication adherence was identified using an unstructured self-report question, “Do you sometimes forget to take your antihypertensive pills?” with responses of “yes” or “no.” This item has been frequently used to detect treatment adherence in older patients with hypertension in China, where forgetting to take pills is a leading contributor to poor adherence [51, 52].

### Covariates

According to previous research [11, 53–55], ten socio-demographic and health-related factors known to affect blood pressure control were included as potential confounders. These covariates included age, gender (female, male), marital status (married, others), live arrangement (live alone, live with others), educational level (less than primary school, junior high school, senior high school and above), residence (rural, urban), co-morbidity included cardiovascular diseases(yes, no) and diabetes (yes, no), smoking status (never, current/former), alcohol intake (yes, no), and self-rated health (very poor/poor/fair, excellent/good). Moreover, for the different dependent variables in different regression models, the confounding factors are controlled corresponding. For the blood pressure control, hypertension duration was controlled [56]. For the depressive symptoms, the number of all comorbidities was controlled [57].

### Statistical analysis

First, we examined the descriptive statistics for all the study variables. The summary statistics for all participants were reported separately by blood pressure control status using proportions for categorical variables and means, and standard deviation for continuous variables conformed to normal distribution. T-tests and chi-square tests were used to assess differences between blood pressure control groups. For variables that do not conform to a normal distribution, we applied median(P25, P75) to conduct descriptive statistics and Mann–Whitney-U test to assess differences between different blood pressure control groups.

Then, a series of multivariable regression models were conducted to estimate the total, direct and indirect effects of relative deprivation on blood pressure control. First, we conducted a binary logistic regression model to examine the total effect of relative deprivation on blood pressure control. Second, the depressive symptoms and medication adherence were subsequently added as covariates to the logistic regression model to examine the attenuation of the association between relative deprivation and blood pressure control. Third, we conducted one linear regression model and one logistic regression, respectively, with depressive symptoms and medication adherence as dependent variables to examine whether relative deprivation affected depression and medication adherence.

Further, a mediation analysis was conducted to assess if depressive symptoms and medication adherence explained the association between relative deprivation and blood pressure control. Given the dichotomous nature of blood pressure control used in this study, we employed a binary logistic model based on the KHB mediation analysis proposed by Karlson, Hom, and Breen [58]. This method is applicable to logistic regression models with multiple mediators allowing the mediators to be binary variables, and allows us to decompose the total and direct effects of relative deprivation on blood pressure control, and the indirect (mediated) effect through depressive symptoms and medication adherence [59]. The KHB method also provides us with a summary of the respective contribution of each of the mediators.

All models were adjusted for ten control variables prescribed. Sampling weights, estimated as the inverse probability of being selected for the survey, were used in all calculations to obtain representative estimates. All data were analyzed using Stata software, version 15 (StataCorp LLC). The significance threshold was *P* = 0.05.

## Results

### Descriptive statistics

Table 1 shows the descriptive statistics for all the study variables used in the analysis. A total of 2382 participants aged 45 years or older were included in this study. The average age in the sample was 65 years old. 61.3% were female, and most were rural respondents(60.7%). Education varied in the sample, and more than half of the sample was less than primary school (59%). In the analyzed sample, 1550 (65.1%) had uncontrolled blood pressure. Univariate analyses showed that gender, education, prefecture on residence, alcohol intake, smoking, comorbidity (diabetes and cardiovascular diseases), and self-rated health status significantly differed across the controlled blood pressure and uncontrolled blood pressure groups (*P* < 0.05).

**Table 1:** Characteristics of the study samples

	Total	Controlled BP	Uncontrolled BP	*p* value
	(*N* = 2382)	(*n* = 832)	(*n* = 1550)	
**Age(years), mean (SD)**	64.9(9.1)	64.7(8.9)	65.1(9.1)	0.226^a^
**Female, n (%)**	1460(61.3)	544(65.4)	916(59.1)	0.003^b^
**Marital status, n (%)**				0.302^b^
Married	1914(80.4)	659(79.2)	1255(81.0)	
Single/divorced/separated/widowed	468(19.6)	173(20.8)	295(19.0)	
**Live arrangement, n (%)**				0.808^b^
Live alone	318(13.3)	719(86.4)	1345(86.8)	
Live with others	2066(86.7)	113(13.6)	205(13.2)	
**Education, n (%)**				0.004^b^
Less than primary school	1406(59.0)	459(55.2)	947(61.1)	
Junior high school	649(27.3)	235(28.2)	414(26.7)	
Senior high school and above	327(13.7)	138(16.6)	189(12.2)	
**Location, n (%)**				< 0.001^b^
Rural	1446(60.7)	447(53.7)	999(64.5)	
Urban	936(39.3)	385(46.3)	551(35.3)	
**Smoking status, n (%)**				0.493^b^
Current/former smoker	601(25.3)	203(24.4)	398(25.7)	
Never	1781(74.7)	629(75.6)	1152(74.3)	
**Alcohol intake, n (%)**				0.005^b^
Yes	496(79.2)	147(17.7)	349(22.5)	
No	1886(20.8)	685(82.3)	1201(77.5)	
**Diabetes, n (%)**	386(16.2)	58(7.0)	328(21.2)	< 0.001^b^
**Cardiovascular diseases, n (%)**	498(20.9)	196(23.6)	302(19.5)	0.02^b^
**No. of comorbidities, n (%)**				0.831^b^
0–1	2065(86.7)	719(86.4)	1346(86.8)	
2–3	295(12.4)	104(12.5)	191(12.3)	
≥ 4	22(0.9)	9(1.1)	13(0.8)	
**Self-rated health status,n (%)**				< 0.001^b^
Very poor/poor/fair	1181(49.5)	366(44.0)	815(52.6)	
Good/excellent	1201(50.4)	466(56.0)	735(47.4)	
**Hypertension duration(years), mean(SD)**	10.8(8.4)	10.4(8.4)	11.1(8.3)	0.053^a^
**Relative deprivation, median (P**_**25**_**,P**_**75**_**)**	0.46(0.21,0.68)	0.41(0.18,0.65)	0.47(0.22,0.69)	0.001^c^
**Depressive symptoms, mean (SD)**	3.33(4.0)	3.13(3.9)	4.13(4.5)	< 0.001^a^
**Non-adherence, n (%)**	1081(45.4)	137(16.5)	944(61.0)	< 0.001^b^

*SD* Standard Deviation, *BP* Blood pressure

^a^ Two-sample t-test

^b^ Pearson's Chi-square test

^c^ Mann–Whitney-U test

Significant differences were also observed in relative deprivation, depressive symptoms, and medication adherence. Compared with participants with controlled blood pressure, those with uncontrolled blood pressure had higher relative deprivation score (median (P25,P75), 0.47(0.22,0.69) vs 0.41(0.18,0.65), *P* = 0.001), more severe depressive symptoms (mean (SD), 4.13(4.5) vs 3.13(3.9), *P* < 0.001), and poorer medication adherence (61.0% vs 16.5%, *P* < 0.001).

### Association of relative deprivation with blood pressure control, depressive symptoms, and medication adherence

Table 2 shows the association of relative deprivation with uncontrolled blood pressure, high depressive symptoms, and poor medication adherence. Model 1 presents the total effect of relative deprivation on uncontrolled blood pressure. After adjusting for age, sex, marital status, live arrangement, education, equivalized household income, prefecture on residence, alcohol intake, smoking, comorbidity, self-rated health status and hypertension during, an increased relative deprivation index was likely to have higher odds of uncontrolled blood pressure (odds ratio (OR): 2.73, 95% confidence interval (CI): 1.02–7.32). For every one unit increase in the relative deprivation index, the odds of uncontrolled blood pressure increased by 2.73 times. Taken together, relative deprivation was harmful to blood pressure control in middle-aged and older hypertension patients, supporting Hypothesis 1 in our study. Model 2 shows the direct effects of relative deprivation, depressive symptoms, and medication adherence on blood pressure control. After adding depressive symptoms and medication adherence into the model, the significant association between relative deprivation and blood pressure control remained but was slightly attenuated (OR: 2.35, 95%CI: 1.78–7.14), suggesting the partial mediating effect of depressive symptoms and medication adherence on the relationship between relative deprivation and uncontrolled blood pressure. Moreover, higher depressive symptoms (OR: 1.02, 95%CI: 1.01–1.08) and poor medication adherence (OR: 8.85, 95%CI: 7.11–11.05) were both associated with a higher risk of uncontrolled blood pressure.

**Table 2:** The associations of relative deprivation with uncontrolled blood pressure, high depressive symptoms, and non-adherence

	Model 1	Model 2	Model 3	Model 4
	(Blood pressure control)	(Blood pressure control)	(Depressive symptoms)	(Medication adherence)
	OR (95%CI)	OR (95%CI)	β (95%CI)	OR (95%CI)
Relative deprivation	2.73(1.02–7.32)^*^	2.35(1.78–7.14)^*^	2.13(0.41–3.88)^*^	2.11(1.98–2.81)^*^^*^
Age	1.00(0.99–1.01)	1.01(0.99–1.02)	-0.12(-0.03–0.01)	1.12(1.11–1.14)^***^
Female (ref = male)	1.59(1.20–2.10)^**^	1.64(1.20–2.25)^**^	1.03(0.56–1.51)^***^	0.55(0.41–0.75)^***^
Married (ref = others)	1.25(0.92–1.69)	0.17(0.83–1.63)	-0.15(-0.55–0.52)	0.56(0.38–0.83)^**^
Live alone (ref = live with others)	1.12(0.79–1.58)	1.01(0.69–1.48)	0.96(0.35–1.57)^**^	1.02(0.65–1.59)
Education (ref = Senior high school and above)				
less than primary school	1.65(1.23–2.23)^**^	1.91(1.36–2.67)^***^	0.52(0.02–1.05)^*^	1.21(0.87–1.69)
Junior high school	1.33(0.99–1.78)	1.35(0.97–1.89)	0.07(-0.45–0.59)	1.11(0.79–1.52)
Rural residence (ref = urban)	1.52(1.25–1.85)^***^	1.39(1.12–1.73)^**^	0.03(-0.31–0.38)	1.39(1.16–1.67)^***^
Current/former smoker (ref = never)	1.42(1.06–1.90)^*^	1.48(1.01–1.91)^*^	0.38(-0.11–0.88)	2.80(2.23–3.52)^***^
Alcohol intake (ref = no)	1.36(1.03–1.79)^*^	1.23(0.91–1.66)	0.39(-0.08–0.86)	1.08(0.79–1.47)
Diabetes (ref = no)	3.87(2.86–5.23)^***^	4.46(3.24–6.15)^***^	-0.14(-0.56–0.29)	1.21(0.91–1.59)
Cardiovascular diseases (ref = no)	1.48(1.19–1.86)^**^	1.42(1.10–1.83)^*^^*^	0.42(-0.03–0.86)	1.04(0.79–1.36)
Very poor/poor/fair health status (ref = Good/excellent)	1.26(1.05–1.51)^*^	1.35(1.10–1.66)^**^	-1.79(-2.11–-1.47)^***^	1.33(1.08–1.64)^**^
Hypertension duration	1.01(0.99–1.02)	1.02(1.01–1.04)^**^	-	
No. of comorbidities (ref = 0–1)	-	-		-
2–3	-	-	1.03(0.51–1.57)^***^	-
≥ 4	-	-	2.55(0.90–4.20)^**^	-
Depressive symptoms		1.02(1.01–1.08)^*^	-	1.04(1.01–1.07)^**^
Medication adherence (ref = non-adherence)		8.85(7.11–11.05)^***^	-	-

*OR* Odds ratio, *95%CI* 95% confidence interval, β Unstandardized regression coefficient

^*^p < 0.05

^**^p < 0.01

^***^p < 0.001

Model 3 and Model 4 show that relative deprivation was significantly associated with depressive symptoms and poor medication adherence. Specifically, after controlling the covariates in Model 3 (linear regression model), relative deprivation is positively related to depressive symptoms (β: 2.13, *P* < 0.05). In Model 4, after controlling the covariates, relative deprivation was associated with high risks of having poor medication adherence (OR: 2.11, 95%CI: 1.98–2.81). Moreover, depressive symptoms increased the odds of poor medication adherence(OR: 1.04, 95%CI: 1.01–1.07). The multicollinearity test was conducted, and there was no multicollinearity in these four models.

### The mediating roles of depressive symptoms and medication adherence

Table 3 present the KHB mediation analysis of relative deprivation on blood pressure control through depressive symptoms and medication adherence. All models have adjusted for age, gender, education, equivalized household income, location, alcohol intake, smoking, comorbidity, and self-rated health status. First, the table shows the total effect of relative deprivation on uncontrolled blood pressure. This is followed by the decomposed direct and indirect effect of relative deprivation on uncontrolled blood pressure through depressive symptoms and medication adherence. The total effect of relative deprivation on uncontrolled blood pressure was 1.77 (95%CI: 1.28–2.43). There remained a direct effect of relative deprivation on uncontrolled blood pressure independent of the potential mediators (OR: 1.38,95%CI: 1.03–1.96). The indirect effect of relative deprivation on uncontrolled blood pressure through depressive symptoms and medication adherence was 1.28 (95%CI: 1.10–1.39), indicating that depressive symptoms and medication adherence explain some of the association between relative deprivation on blood pressure control, supporting Hypothesis 2 in our study.

**Table 3:** Mediation analysis of depressive symptoms and medication adherence on the association between relative deprivation and blood pressure control

Decomposition of effects	OR (95%CI)	*P* value
Total effect of relative deprivation on uncontrolled blood pressure	1.77(1.28–2.43)	0.001
Direct effect of relative deprivation on uncontrolled blood pressure	1.38(1.03–1.96)	0.031
Indirect effect of relative deprivation on uncontrolled blood pressure through depressive symptoms and medication adherence	1.28(1.10–1.39)	< 0.001
Summary of mediation	%	
Percent of total effect due to both depressive symptoms and medication adherence	43.67%	
Percent of total effect due to depressive symptoms alone	5.91%	
Percent of total effect due to medication adherence alone	37.76%	

All calculated effects account for age, gender, education, income, location, smoking, alcohol intake, comorbidity, and self-rated health

*OR* Odds ratio

The KHB method summarizes the mediation effect due to depressive symptoms and medication adherence at the end of Table 3. 43.67% of the total effect was mediated. Of the total effect of relative deprivation on uncontrolled blood pressure, 5.91% of the total effect was due to depressive symptoms alone, and 37.76% of the total effect was due to medication adherence alone.

## Discussion

This study examined the role of relative deprivation on blood pressure control in middle-aged and older Chinese adults with hypertension. Specifically, we examined whether relative deprivation increased the odds of uncontrolled blood pressure, and whether depressive symptoms and medication adherence could partly explain this. Our findings indicated that higher relative deprivation coincides with higher odds of uncontrolled blood pressure, supporting our first hypothesis. Furthermore, depressive symptoms and medication adherence explained nearly half of the effect of relative deprivation on blood pressure control, supporting our second hypothesis. This was mostly driven by medication adherence, which accounted for 37.76% of the association between relative deprivation and blood pressure control. Depressive symptoms were also a mediator, explaining 6% of the association between relative deprivation and blood pressure control.

The current study confirmed that the association between relative deprivation and blood pressure control would be partially mediated by depression and medication adherence, possibly revealing the psychosocial and behavioral mechanisms concerning how relative deprivation might indirectly influence blood pressure control. Since relative deprivation is closely connected to income distribution, higher income inequality may trigger emotional discomfort through depressive symptoms [60]. Study evidence has shown the mediating role of depressive symptoms in the link between relative deprivation and cause-specific mortality, self-rated health, suicide risk and quality of life [24, 61, 62]. The depressive symptoms also further threatened health outcomes. Previous research indicated that depressive symptom is one significant risk factor for uncontrolled blood pressure [63]. Therefore, our results confirm that the psychological process provides a critical pathway linking income inequality and health outcomes. Relative deprivation could affect blood pressure control by increasing emotional discomfort (e.g., depressive symptoms).

Moreover, participants with high relative deprivation are more likely to have poor medication adherence, which leads to a higher risk of uncontrolled blood pressure. The relationships between income inequality and unhealthy behaviors such as tobacco use, physical inactivity, obesity have been well demonstrated empirically, as well as poor medication adherence [64–66]. The relatively deprived individuals could become more prone to suboptimal adherence due to an inability to access adequate healthcare (including financial constraints, limited transportation, and inflexible employers, among other challenges) [67]. What’s more, relative deprivation can cause psychosocial stress that harms an individual due to actual or perceived social status, or lack thereof, as well as access to mobility and resources, which could further support a possible explanation for the impact of relative deprivation on medication adherence observed in our study [68]. Furthermore, medication adherence plays a specific and critical role in hypertension control [69]. Therefore, our results suggest that health behavior is a critical pathway linking income inequality and health outcomes. Relative deprivation may affect blood pressure control indirectly via medication adherence. Furthermore, participants with high depressive symptoms also increased the odds of poor medication adherence, this result is consistent with reports from previous studies examining individuals with chronic disease [52, 70]. Potential mechanisms by which depression affects medication adherence might include decrements in memory and cognition and a lack of energy and motivation to continue taking antihypertensive medication [71].

In addition to the psychosocial pathway, and behavioral pathway analyzed in our study, the unique cultural background of China also plays a potential role in the positive association between higher relative deprivation and higher odds of uncontrolled blood pressure. Ceremonies, such as funerals and weddings, are important traditions in Chinese society. People who skip these events or spend less than their more affluent counterparts lose face and risk becoming social isolation [72]. Specifically, the relatively deprived increase spending on funerals and gifts as competition for status intensifies. The relatively deprived families spend a much higher budget on hosting funerals and wedding ceremonies in the face of intensifying local competition for status. Studies have found that gift and festival spending due to relative deprivation feelings have squeezed out basic food and healthcare consumption [73].

Our study indicate the importance of relative deprivation to the blood pressure control among middle-aged and older hypertension patients. These findings are consistent with and extend previous studies reporting associations between socioeconomic context and hypertension outcomes [10, 11, 74, 75]. Furthermore, we contribute to the research on relative deprivation and blood pressure control by demonstrating the mediating roles of depression and medication adherence in the association between relative deprivation and blood pressure control. The mediation analysis could help us understand the mechanism of the association between relative deprivation and blood pressure control, and policies and interventions could be designed based on the mechanism. Our findings uphold that relative deprivation directly and negatively affects blood pressure control while also indirectly affecting blood pressure control by psychological pathway (depressive symptoms) and health-related behavioral pathway (medication adherence) [76, 77]. The results suggest us that active intervention of depression and medication adherence may play an important role in controlling blood pressure in middle-aged and older adults with hypertension. The current study expands the applicability of relative deprivation hypothesis beyond general health to specific areas of chronic disease, as researchers have previously pointed out to be an important area of future investigation.

This study also has several limitations that provide directions for future research. First, the cross-sectional design of the survey is difficult to determine temporal ordering between relative deprivation and uncontrolled blood pressure, which prevents us from making causal relationships between them. Future studies may need to define more pathways of associations between relative deprivation and health outcomes using long-term follow-up data. Second, it is impossible to explain how much relative deprivation is here. The measurement of subjective relative deprivation was absent, so it is unknown whether those with a higher Kakwani Index in fact perceived themselves as relatively deprived. However, using objective relative deprivation can simultaneously prevent common method bias caused by subjective predictors and self-reported outcome measures. Future research relating to the health effects of subjective relative deprivation would be conducted to compare to objective relative deprivation. Third, we did not have data regarding current pharmacological treatment for depression. A side effect of antidepressants was elevated blood pressure, which may be associated with blood pressure control. Forth, using a single item of medication adherence is not accurate enough, although it is simple and general. Meanwhile, the antihypertensive drug type and quantity were also not collected, which may be the critical covariates of medication adherence. In addition, this survey uses a structured self-reported questionnaire, and the response cannot completely rule out the possibility of recall bias. Last, our study was conducted in a single city, potentially limiting the findings' generalizability.

## Conclusions

In conclusion, individual relative deprivation could threaten blood pressure control among middle-aged and older hypertension patients through the mechanisms of depression and medication adherence. Hence, improving blood pressure control may require more than just health management and education but fundamental reform of the income distribution and social security system to narrow the income gap, reducing relative economic deprivation. Additionally, interventions tailoring psychological services and medication adherence could be designed to reduce the harmful effect of relative deprivation on blood pressure control among disadvantaged individuals. Finally, as government implements strategies to improve blood pressure control, it is essential to account for the potential health risks associated with relative deprivation to ensure that the care we deliver improves outcomes for all populations.

## Data Availability

The datasets used and/or analysed during the current study are available from the corresponding author on reasonable request.

## Abbreviations

BP: Blood pressure
SD: Standard deviation
OR: Odds ratio
95%CI: 95% Confidence interval
